# Staged Reconstruction and Salvage of a Small Finger With a Corticocancellous Distal Radius Autograft: A Case Report

**DOI:** 10.7759/cureus.91256

**Published:** 2025-08-29

**Authors:** Emmanuel Franco, Zayd Ayas, Frank Gerold

**Affiliations:** 1 Orthopaedic Surgery, University of Texas Rio Grande Valley School of Medicine, Edinburg, USA

**Keywords:** corticocancellous autograft, degloving, hand surgery, orthopedic surgery, phalanx fracture, staged reconstruction, trauma

## Abstract

Finger reconstruction in the aftermath of severe trauma poses significant challenges in restorative surgery, particularly when addressing extensive soft tissue damage, comminuted fractures, and simultaneous neurovascular complications. This study presents a case of a patient with a complicated proximal phalangeal fracture who pursued digit salvage.

A 31-year-old Hispanic woman sustained severe injury to her right fifth digit during an all-terrain vehicle (ATV) rollover resulting in an exposed, comminuted fracture of the proximal phalanx with extensive structural and neurovascular compromise. She pursued digit salvage despite a high risk of failure and complication. The patient underwent four operations over a six-month period, including a distal radius corticocancellous autograft placement.

Through multiple surgeries, we were successfully able to salvage the finger, resolve the patient’s pain, and provide her with an aesthetically pleasing, functional result. This illustrates the importance of patient involvement in decision-making every step of the way.

## Introduction

Phalangeal fractures are the most common fractures of the hand, accounting for 18% of all upper extremity fractures [1]. Furthering our understanding of the management of different types of phalangeal fractures is essential given the complexity of the surrounding joint compartments, tendons, and intricate neurovascular supply. Repair of any phalangeal fracture aims to preserve the remaining neurovascular supply, minimize further soft tissue injury, and allow for mobilization of a sensate digit with preserved length as soon as stability is achieved [2].

Treatment of open fractures associated with joint nonunion and significant tissue loss is generally not advised [3]. This case discusses the presentation, treatment, and outcomes of a patient who presented with extensive bone, tendon, nerve, and artery damage who was a good candidate for amputation but elected to undergo digit salvage and reconstruction procedures.

## Case presentation

**Figure 1:**
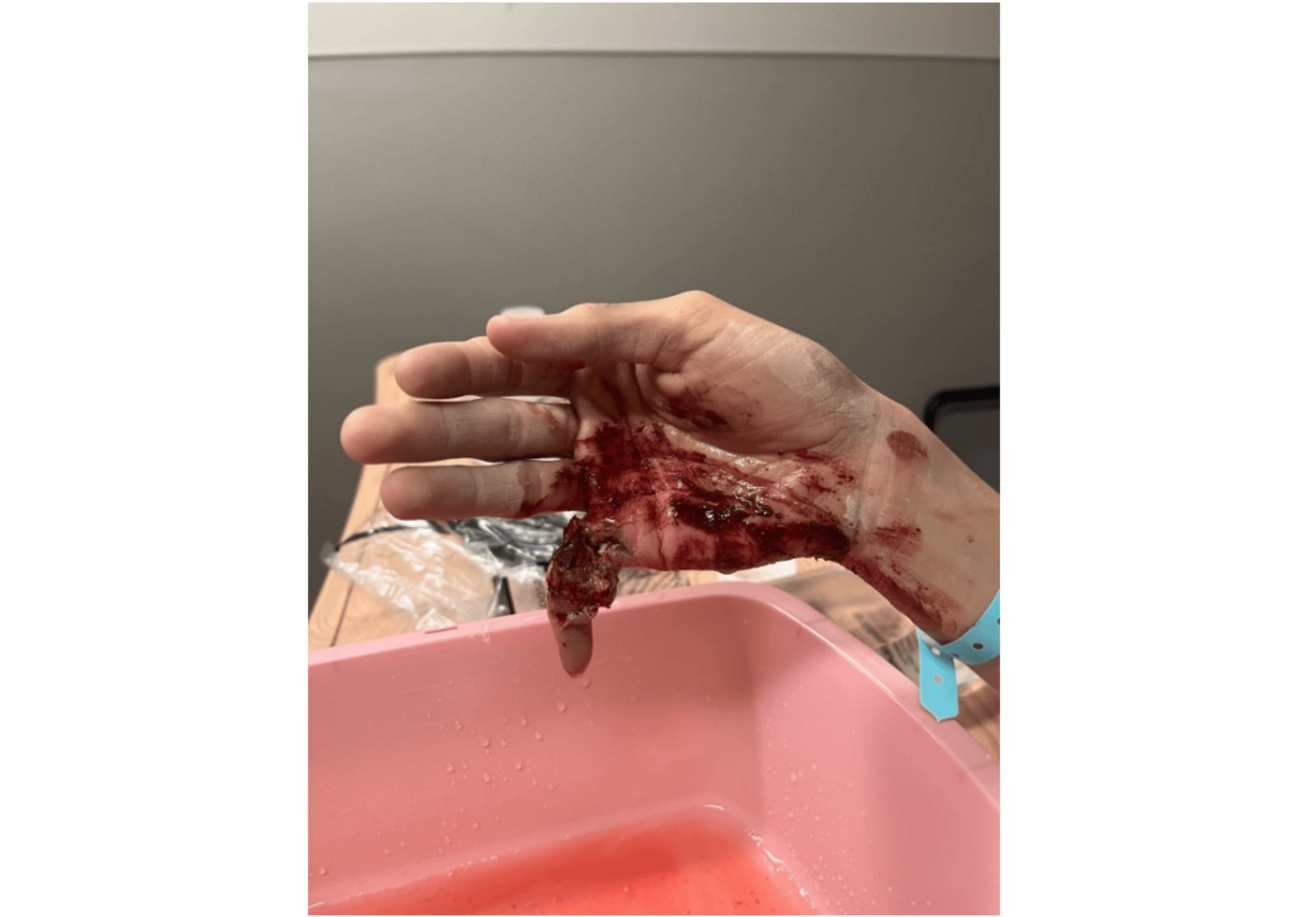
Initial Presentation Image of right hand at initial presentation following severe all-terrain vehicle (ATV) degloving injury to the right small digit showing exposed, comminuted fracture of proximal phalanx and vascular injury.

A 31-year-old female patient who presented to the emergency department after having injured the small digit of her right hand during an all-terrain vehicle (ATV) rollover accident while a restrained passenger traveling at 20 mph. She denied loss of consciousness or any concurrent injuries. Additionally, she denied any relevant medical, family, or psychosocial history. Physical exam revealed an exposed proximal phalanx of a deformed small digit, grossly contaminated with dried mud and resting in extension (Figure 1).

Sensation to the ulnar side of the pulp was intact, however the remaining soft tissue on the distal radial aspect of the finger was insensate. Capillary refill, though delayed, was present at the tip of the finger. Right-hand X-rays showed a comminuted fracture of the proximal phalanx of the fifth digit with likely bone loss.

**Figure 2:**
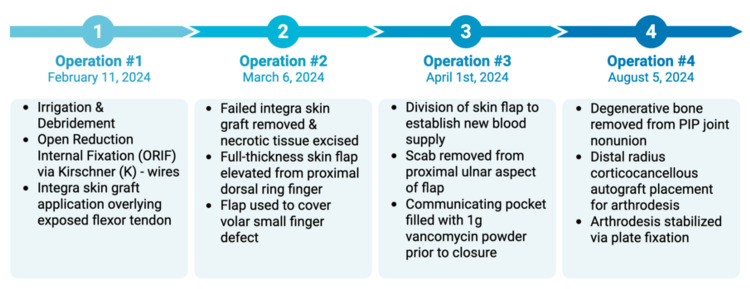
Timeline of Staged Reconstruction A total of four operations ensued within the time span of six months, all of which have been summarized in this timeline. PIP: proximal interphalangeal

It was reasoned through exam findings and assessment that, due to extensive skin and bone loss, as well as injuries to flexor tendon, radial nerve, and artery, digital amputation would be a good option, allowing her to return sooner to normal activities. Salvage procedures could likely result in post-operative complications such as infection and non-union, especially given the high level of contamination present. Nonetheless, the patient insisted on proceeding with a salvage operation, voicing understanding of the high possibility of failure and likelihood for future amputation. A timeline of the staged reconstruction that followed has been provided (Figure 2).

**Figure 3:**
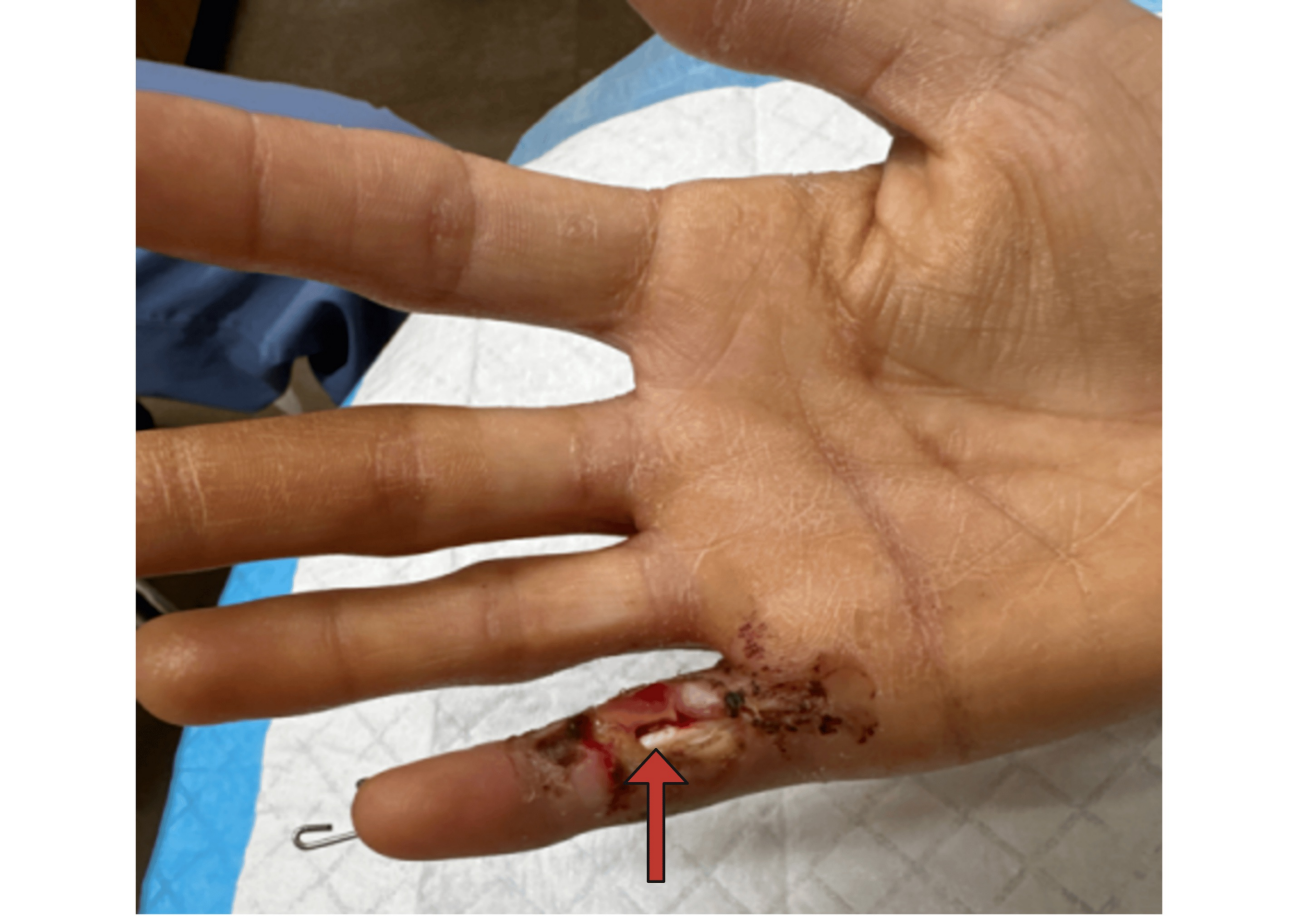
Exposed Flexor Tendon The small digit flexor tendon (red arrow) became exposed following placement of the Integra skin graft.

The first operation was performed that same day and consisted of extensive irrigation and debridement of the open fracture, open reduction internal fixation (ORIF) of the proximal phalanx fracture using two anterograde-placed 0.9mm Kirschner (K)-wires extending into the distal metacarpal, and application of an Integra skin graft (Integra LifeSciences, Princeton, NJ, USA) over the salvageable flexor tendon (Figure 3).

**Figure 4:**
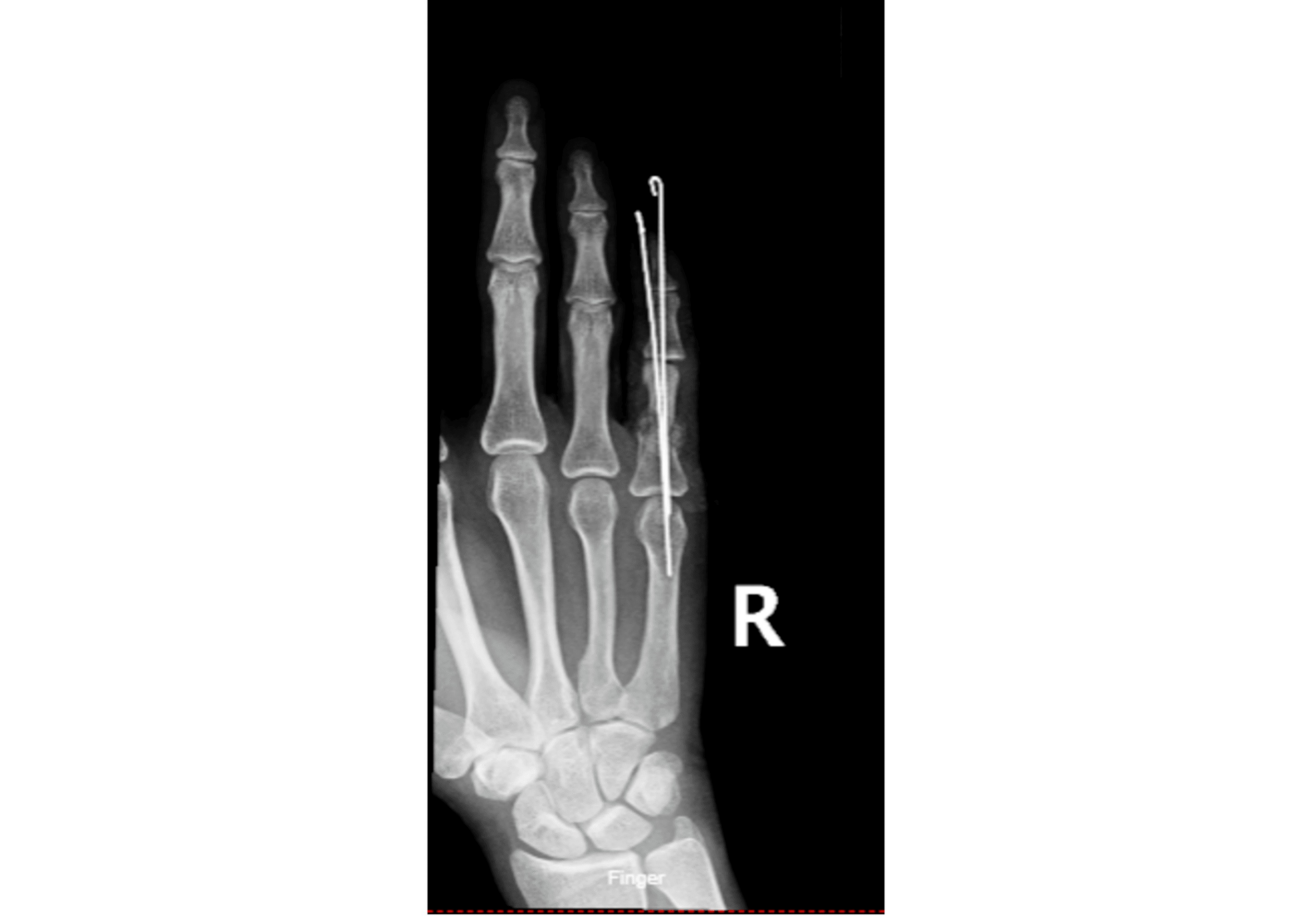
ORIF During First Operation PA X-ray demonstrating open reduction internal fixation (ORIF) of right small digit using two anterograde-placed K-wires

Throughout the procedure, suction curette tips, dental picks, and small tenotomy scissors were used to remove devitalized skin, tissue, and bone. Adequate reduction and stable fixation were confirmed via fluoroscopy (Figure 4).

A mixture of 1 g crushed cancellous bone allograft and 1 g vancomycin powder was used to fill the void where the volar phalanx had been. The entire finger was pink at the end of the procedure, wherein Xeroform, bulky gauze dressings, a digital nerve block, and an ulnar gutter splint were placed. The patient was prescribed broad-spectrum antibiotics and instructed not to move the finger until follow-up.

**Figure 5:**
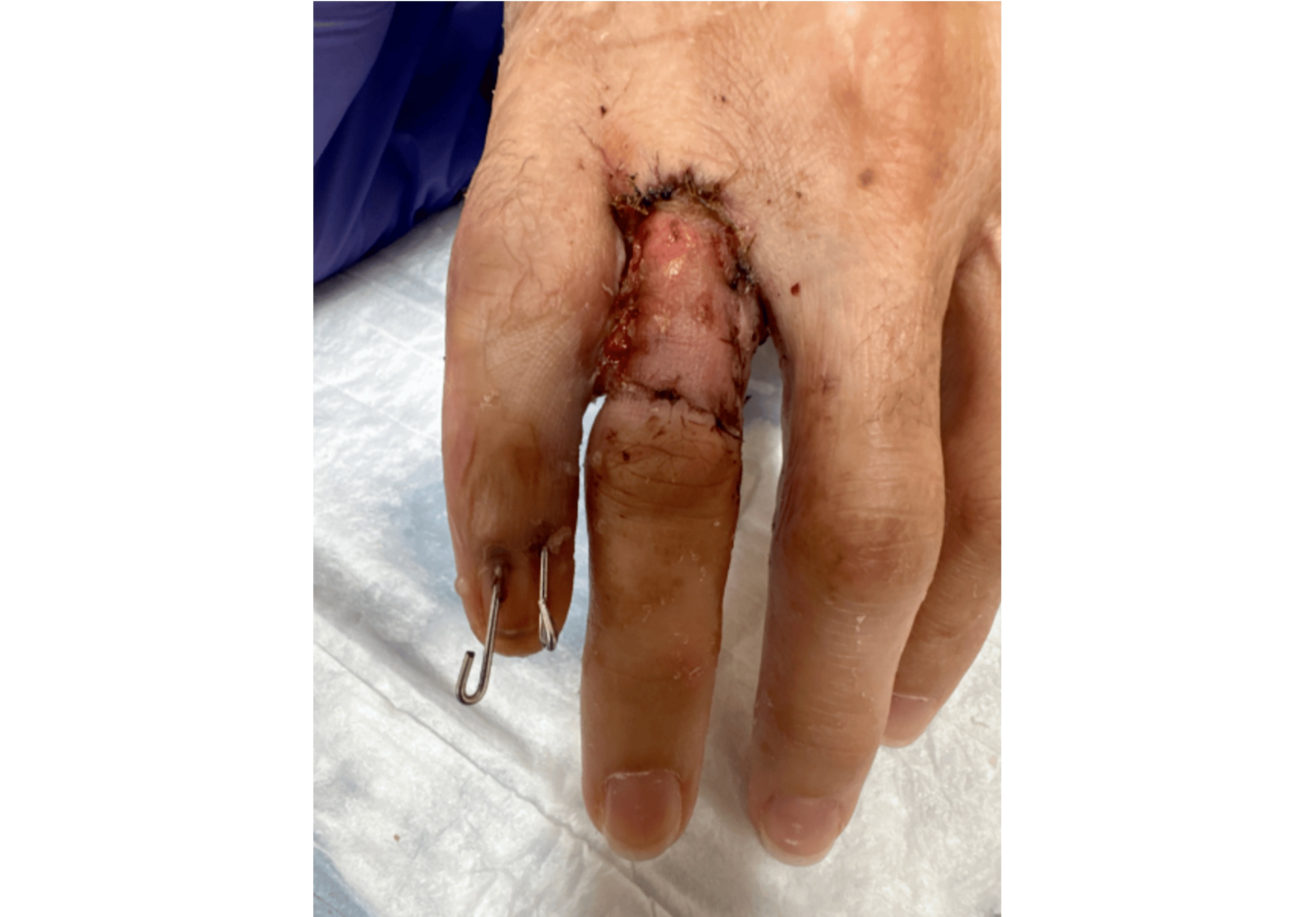
Cross-Finger Flap Donor Site Right ring finger cross-finger flap coverage donor site five days following procedure showing adequate tissue healing.

Three weeks following the initial operation, it was noted that the Integra skin graft did not take; the patient elected for a cross-finger flap coverage of the exposed flexor tendon. After excising necrotic skin, irrigating thoroughly, and confirming lack of infection, a rectangular, full-thickness skin flap was elevated from the proximal dorsal aspect of the right ring finger, which was used to cover the volar small finger defect (Figure 5).

**Figure 6:**
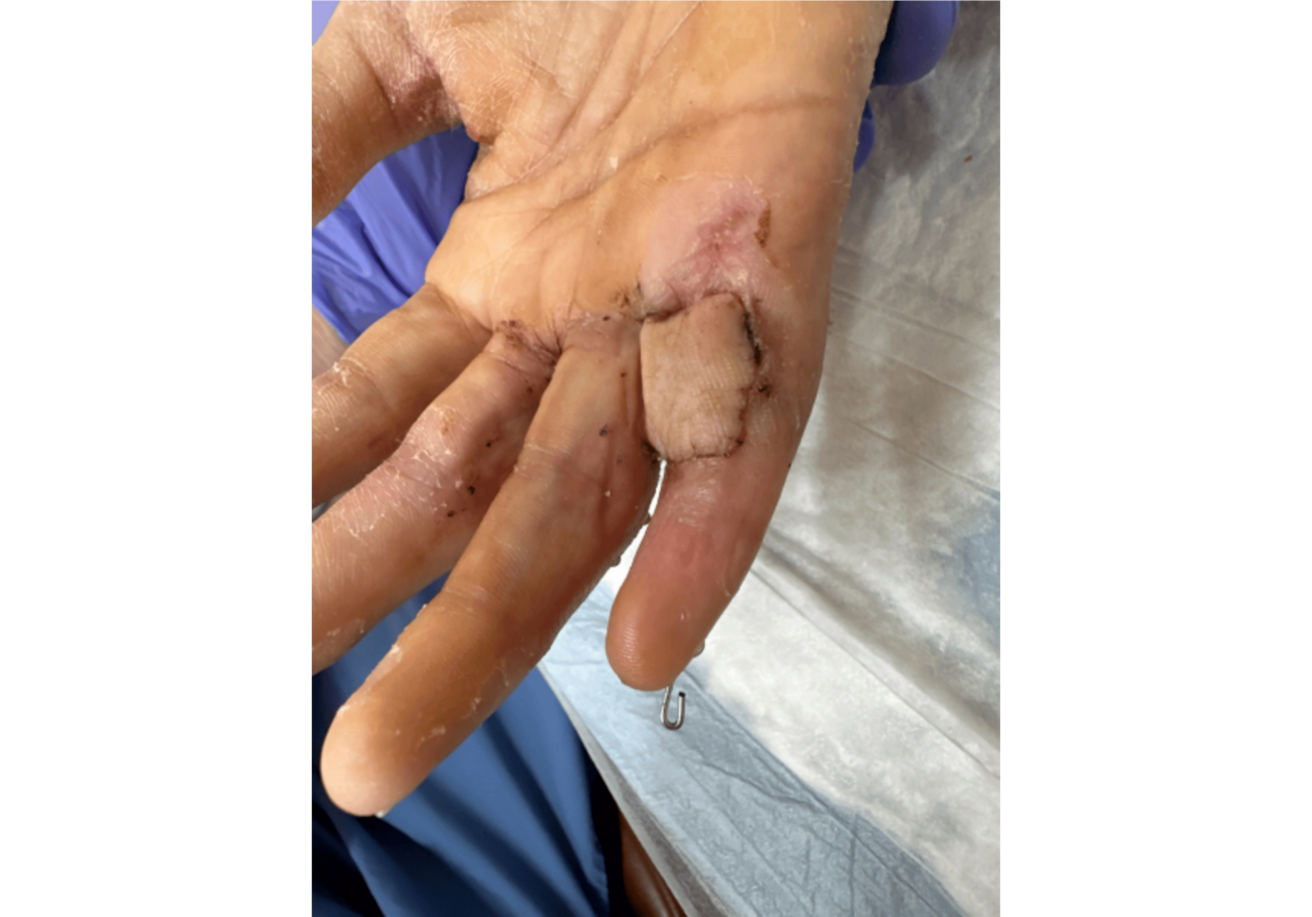
Cross-Finger Flap Recipient Site Right ring finger cross-finger flap coverage recipient site five days following procedure showing adequate tissue healing.

Excellent bleeding and capillary refill were noted at both the flap donor and recipient sites (Figure 6).

**Figure 7:**
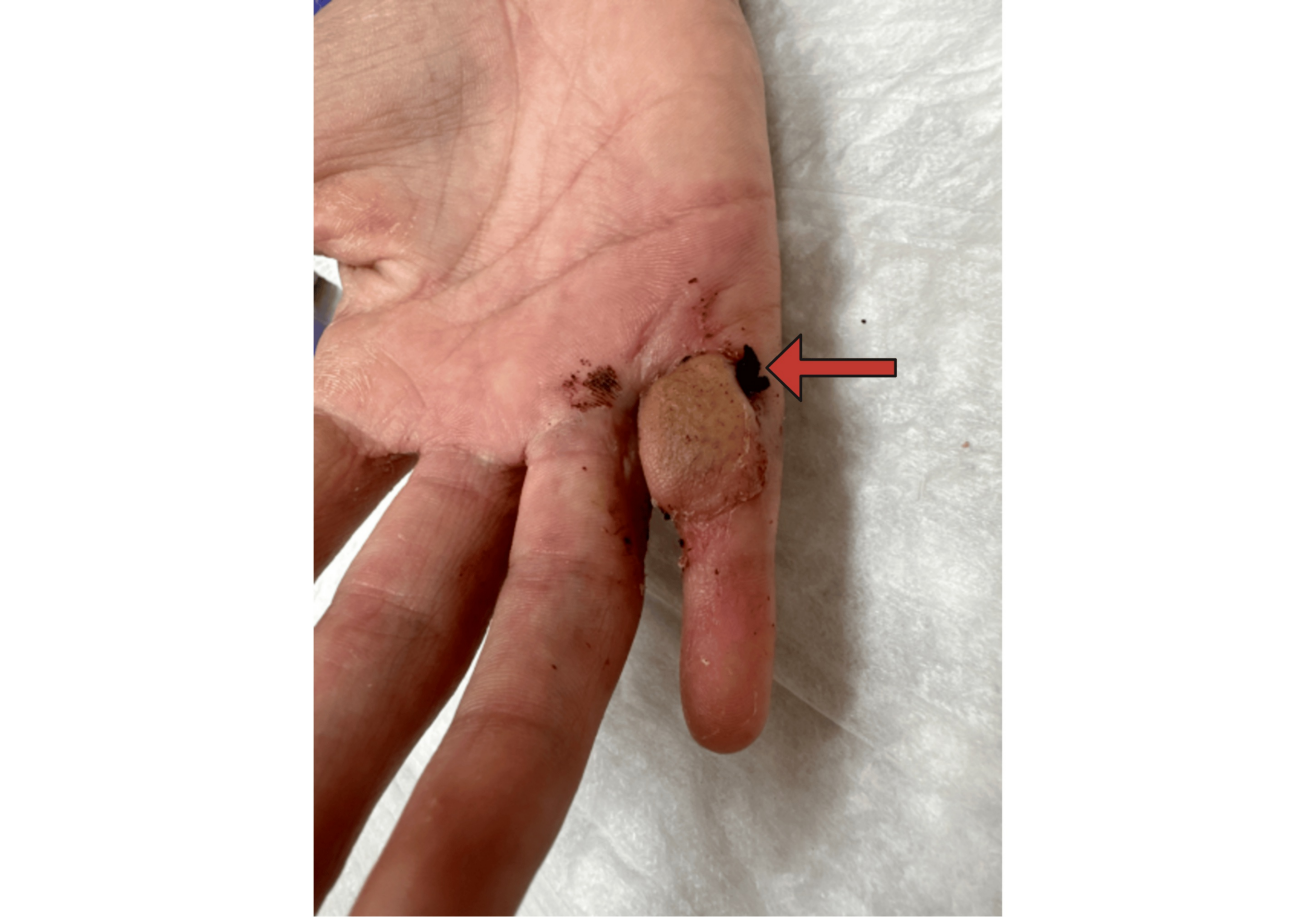
Minor Flap Complication Cross-finger flap coverage, although successful, resulted in a deep pocket lined by devitalized skin (red arrow).

The third operation occurred four weeks after the second procedure. This consisted of division of the pink, viable skin flap on the small finger, only complicated by a small yet deep opening noticed after removing a scab on the proximal, ulnar aspect of the flap (Figure 7).

This opening was filled with a single dose of 1 g vancomycin powder and closed with a single gut suture.

**Figure 8:**
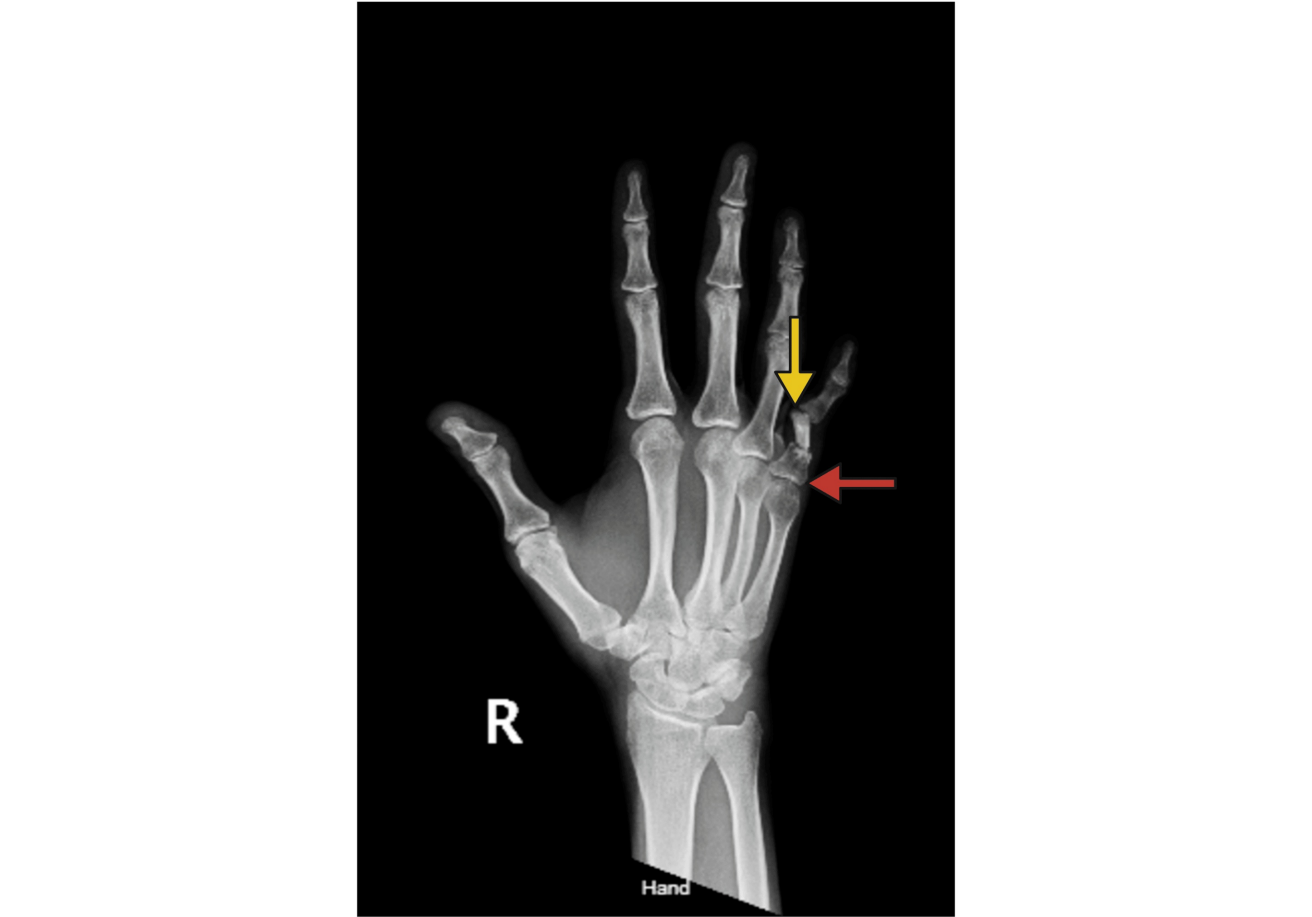
Simultaneous MCP Joint Malunion and PIP Joint Nonunion PA X-ray showing metacarpophalangeal (MCP) joint malunion (red arrow), degenerative proximal phalanx, and proximal interphalangeal (PIP) joint nonunion (yellow arrow).

The last operation, completed four months after the skin flap division, was indicated due to simultaneous tender proximal phalanx malunion and proximal interphalangeal (PIP) joint nonunion, as evidenced by fluoroscopy (Figure 8).

Initially gaining access through a dorsal incision, the extensor tendon was divided midline, along with the dorsal capsule and collateral ligaments. This allowed for optimal visualization of the PIP joint, after which degenerative bone was removed with a Rongeur from both the base of the middle phalanx and the head of the proximal phalanx, leaving a 1 cm long gap.

After irrigating generously, an appropriately-sized corticocancellous autograft was harvested from the ipsilateral distal radius, immediately ulnar to Lister’s tubercle, using a 1.1mm K-wire to mark a peppered perimeter and an osteotome to chisel and elevate the bony graft. Retracting the extensor pollicis longus (EPL) tendon radially after opening its sheath was an essential step in preserving dorsal wrist compartments. The donor site defect was filled with 2 g demineralized bone matrix (DBM) putty and the extensor retinaculum was closed lying over the transposed EPL.

**Figure 9:**
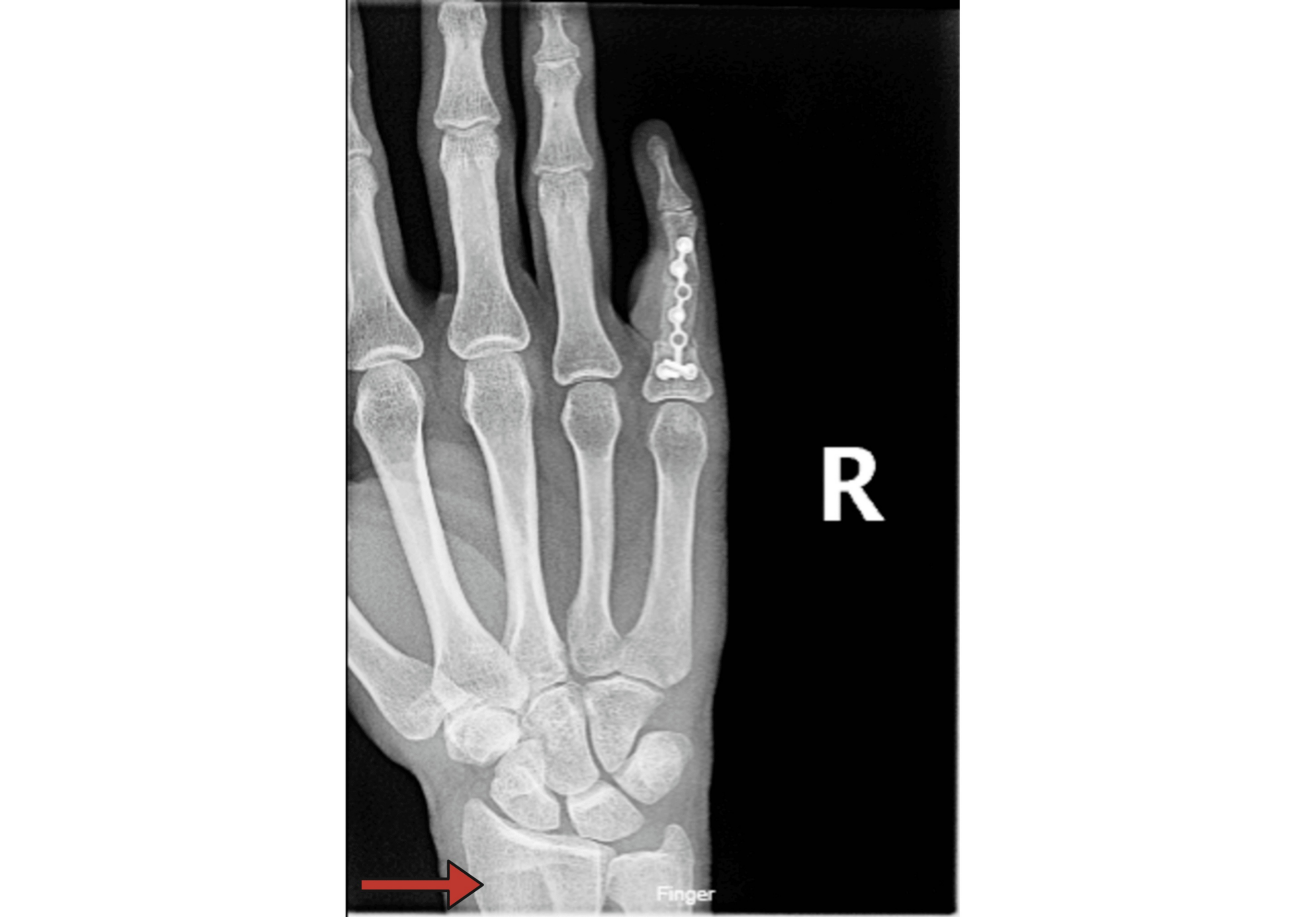
Status Post PIP Joint Arthrodesis PA X-ray taken one week after distal radius autograft fixation via proximal interphalangeal (PIP) joint arthrodesis and a 1.5mm plate. The corticocancellous donor site is visible toward the distal end of the radius (red arrow).

The corticocancellous block was wedged in between the remaining proximal phalanx and the PIP arthrodesis site distally. The bone graft was contoured further to achieve adequate opposition and slight flexion at the arthrodesis, before a 1.5mm plate was placed and fixed with cortical screws (Figure 9).

The operative site was irrigated, 1 g vancomycin powder was applied, and the incision was closed. A well-padded volar resting splint was applied, and the patient was instructed not to move the finger or bear any weight.

**Figure 10:**
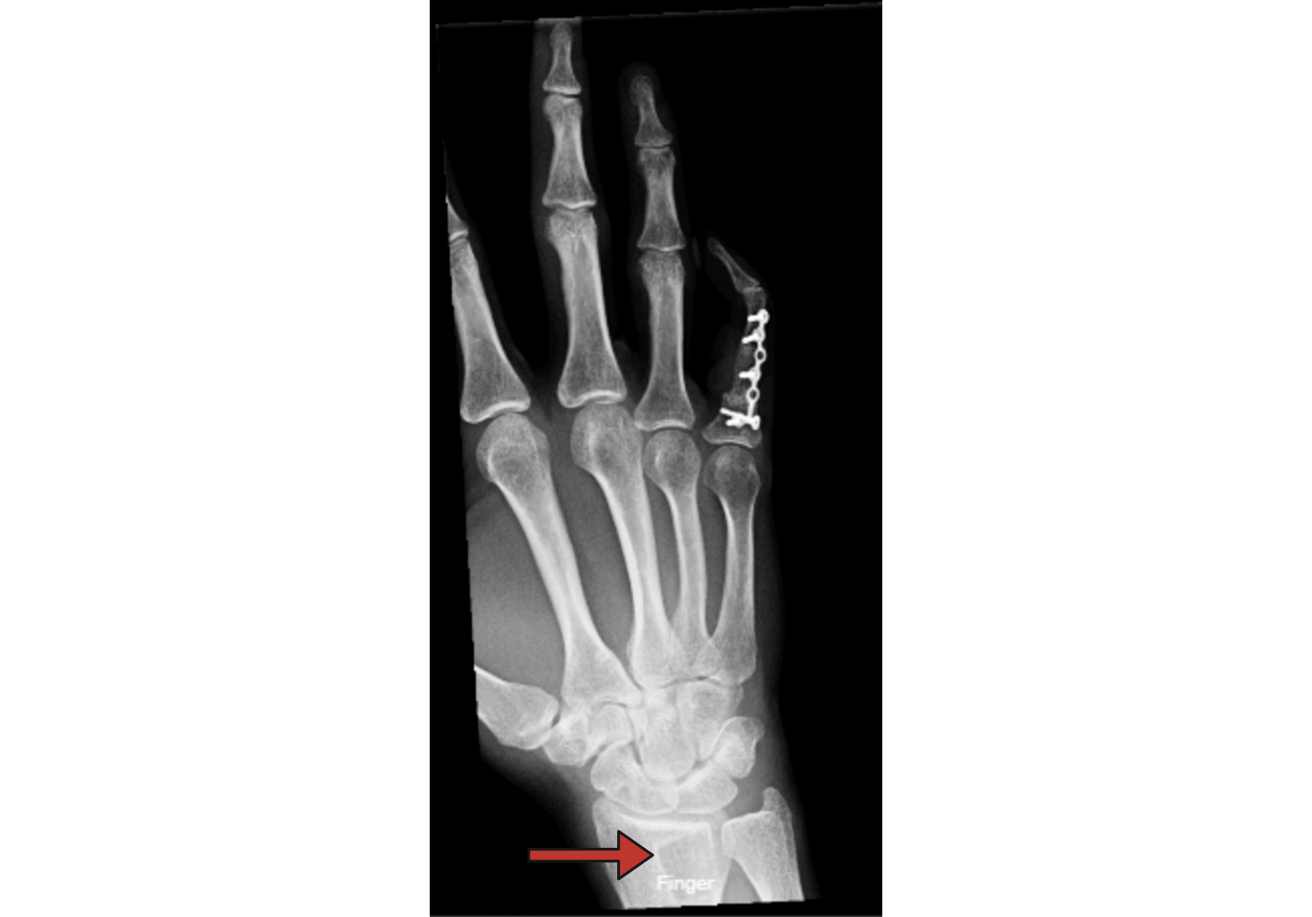
Three Month Follow-up Visit PA X-ray taken three months after distal radius autograft fixation via proximal interphalangeal (PIP) arthrodesis and a 1.5mm plate. The corticocancellous donor site is visible toward the distal end of the radius with continued healing (red arrow).

At her three-month postoperative follow-up visit, our patient reported complete satisfaction with both the aesthetic result and remaining functionality (Figure 10).

Sensation and vascularity were intact throughout the digit and metacarpophalangeal (MCP) joint flexion at the time reached 70 degrees, almost allowing her to make a closed fist. Identical findings were reported at the patient's subsequent follow-up encounter three months after this initial follow-up visit. The patient has expressed feeling complete certainty that the correct decision was made in deciding to pursue salvage and staged reconstruction of her finger. She reports being especially pleased with the aesthetic result and semblance to her contralateral hand from further physical distance.

## Discussion

Proximal phalangeal fractures of the hand are not only common but also challenging to repair and can result in an increased chance of reinjury and revision surgery. After optimal treatment, there still exists a heightened risk of stiffness, osteomyelitis, and septic joint, potentially indicating arthrosis [4]. One retrospective analysis of 54 patients with phalangeal fractures and vascular injury found that more than half required at least one reoperation, and 10% ended with eventual digit amputation [5]. 

Open fracture dislocations of the PIP joint involving substantial bone loss are further complicated by a scarcity of well-established options for bony defect repair in existing literature, including options for bone grafts and postoperative rehabilitation. 

Our patient had extensive soft tissue damage and bone loss. Considering the high degree of injury and significant contamination in our patient, the situation called for a more emergent approach. Open, nonunion injuries are typically dealt with by immediate aggressive debridement of atrophic or infected soft tissue and bone. Tenolysis and bone graft are then placed to close the bony defect, followed by graft or flap coverage within the next week [3]. Alternatively, phalangeal defects are sometimes purposely planned in staged reconstructions where the soft tissue injury is addressed first before the bone [6]. Given our patient’s condition we opted for the former method.

Isolated arthrodesis with a bony autograft is another option for treating chronic PIP joint malunions [3]. An ideal autograft must aim to maximize anatomical stability and approximate mechanical joint reconstruction, while minimizing donor site complications. The hemi-hamate autograft has been considered a reliable option for reconstruction of acute or chronic PIP joint fracture-dislocations [7]. Drawbacks of this method, however, include difficulty with reconstruction of the proximal phalanx upper lip and resulting donor site instability when compared to the ideal autograft. To optimize this, joint reconstruction using a second toe middle phalanx osteochondral graft was introduced. A case series assessing this option found no complaints of joint instability requiring revisional surgeries following this operation in 11 patients, making this a seemingly reliable method [8].

One isolated case used a cylindrical osteochondral plug, harvested from a non-weight-bearing portion of the lateral condyle of the knee, to press fit onto a same-sized drill hole made by the surgeon, just inferior to the PIP joint [9]. Although the patient had no complaints of pain or range of motion (ROM) limitation in either the donor or recipient sites, this injury involved significantly less surrounding soft tissue and neurovascular insult than in our patient, limiting its relevance in the present case.

Finally, a classic case series described and analyzed postoperative outcomes of 14 patients undergoing proximal phalangeal reconstruction after PIP fracture dislocation using a distal radius corticocancellous graft [10]. Here, k-wires were used to immobilize the graft. In our patient, we decided to fix the graft via plates and screws, which are typically used in more unstable phalangeal fractures to be able to achieve primary bone healing [2]. This study reported an average improvement in PIP ROM from 30 degrees to 68 degrees, with the only negative outcomes being a slightly reduced joint space and enlarged volar lip.

Given the dearth of large-scale studies reporting outcomes of corticocancellous autograft options, as well as the patient course in this present case, it is the authors' opinion that a distal radius autograft should be considered a reasonable option for reconstruction of a proximal phalanx following traumatic degloving injuries of similar patterns. This method, when undertaken alongside adequate soft tissue preservation and coverage, is able to provide a sensate digit of original length that patients may be aesthetically satisfied with. Nonetheless, patient counseling and realistic goal setting are uniquely paramount here, considering the possible lengthy timeline necessary.

## Conclusions

Proximal phalangeal fractures are a challenging issue in hand surgery, particularly when complicated by extensive soft tissue damage. The operative and rehabilitative course of this patient case demonstrates a distal radius allograft as a feasible reconstructive option for complex proximal phalangeal fractures, given adequate repair of concomitant neurovascular and tissue injury. It also highlights the importance of frequent conversation with patients in furthering their informed decision-making capacity.​
